# Phosphorylation of Sox2 at Threonine 116 is a Potential Marker to Identify a Subset of Breast Cancer Cells with High Tumorigenecity and Stem-Like Features

**DOI:** 10.3390/cancers10020041

**Published:** 2018-02-03

**Authors:** Nidhi Gupta, Keshav Gopal, Chengsheng Wu, Abdulraheem Alshareef, Alexandra Chow, Fang Wu, Peng Wang, Xiaoxia Ye, Gilbert Bigras, Raymond Lai

**Affiliations:** 1 Department of Laboratory Medicine and Pathology, Cross Cancer Institute, University of Alberta, 11560 University Avenue, Edmonton, AB T6G 1Z2, Canada; nidhi2@ualberta.ca (N.G.); gopal@ualberta.ca (K.G.); chengshe@ualberta.ca (C.W.); al15@ualberta.ca (A.A.); alexchow1@hotmail.com (A.C.); fw2@ualberta.ca (F.W.); pw2@ualberta.ca (P.W.); xiaoxia@ualberta.ca (X.Y.); gilbert.bigras@albertahealthservices.ca (G.B.); 2 Department of Oncology, University of Alberta, Edmonton, AB T6G 1Z2, Canada; 3 DynaLIFE_DX_ Medical Laboratories, Edmonton, AB T6G 1Z2, Canada

**Keywords:** breast cancer, intra-tumoral heterogeneity, Sox2, phosphorylation, immunohistochemistry

## Abstract

We have previously identified a novel phenotypic dichotomy in breast cancer (BC) based on the response to a SRR2 (Sox2 regulatory region 2) reporter, with reporter responsive (RR) cells being more tumorigenic/stem-like than reporter unresponsive (RU) cells. Since the expression level of Sox2 is comparable between the two cell subsets, we hypothesized that post-translational modifications of Sox2 contribute to their differential reporter response and phenotypic differences. By liquid chromatography-mass spectrometry, we found Sox2 to be phosphorylated in RR but not RU cells. Threonine 116 is an important phosphorylation site, since transfection of the T116A mutant into RR cells significantly decreased the SRR2 reporter luciferase activity and the RR-associated phenotype. Oxidative stress-induced conversion of RU into RR cells was accompanied by Sox2 phosphorylation at T116 and increased Sox2-DNA binding. In a cohort of BC, we found significant correlations between the proportion of tumor cells immuno-reactive with anti-phosphorylated Sox2^T116^ and a high tumor grade (*p* = 0.006), vascular invasion (*p* = 0.001) and estrogen receptor expression (*p* = 0.032). In conclusion, our data suggests that phosphorylation of Sox2^T116^ contributes to the tumorigenic/stem-like features in RR cells. Detection of phospho-Sox2^T116^ may be useful in identifying a small subset of tumor cells carrying stem-like/tumorigenic features in BC.

## 1. Introduction

Intra-tumoral heterogeneity is a well-recognized phenomenon in cancer biology, and its clinical significance is highlighted by the existence of cancer stem cells (CSCs), a small population of cancer cells believed to be a major contributory factor to chemo-resistance and disease relapses [1]. Breast cancer (BC) is the first solid tumor in which CSCs are identified [2]. CSCs characterized by the CD44^high^/CD24^low^ immunophenotype are found to have high levels of self-renewal, chemo-resistance and tumorigenecity [2,3]. CSCs also have been described in many other cancer types, and the identification of CSCs is often dependent on their expression of specific cell surface markers such as CD133 and CD49f [3,4] and less commonly, the enzymatic activity of specific cytoplasmic proteins such as aldehyde dehydrogenase 1 [5]. In addition to the ‘classic’ CSCs, others and we have identified small subsets of BC cells that have relatively high levels of cancer stem-like features and tumorigenicity, and these cells can be detected and purified based on their responsiveness to specific transcription factor reporters [6,7,8]. Specifically, using a commercially available reporter designed to detect the transcription activity of Sox2, an embryonic stem cell marker, we have shown that reporter responsive (RR) cells are more stem-like and tumorigenic than those that are reporter unresponsive (RU) in the estrogen receptor-positive BC as well as the triple-negative BC models [8,9,10]. In MCF7 and ZR751, two estrogen receptor-positive BC cell lines, Sox2 is directly implicated in conferring the cancer stem-like features in RR cells, since siRNA knockdown of Sox2 in these cells was found to significantly decrease these properties.

While Sox2 is an important factor to the RR phenotype in estrogen receptor-positive BC cells, how the RU/RR dichotomy is generated is incompletely understood. In MCF7 and ZR751 cells, Sox2 was found to be expressed relatively abundantly in both RU and RR cells [8]. Thus, the RU/RR dichotomy cannot be attributed to a differential Sox2 protein expression level between the two cell subsets. Since Sox2 is a transcription factor and expected to exert its biological effects in the nuclei, we also asked if there is a substantial difference in the nuclear localization of Sox2 between RU and RR cells. In this regard, we did not identify evidence in support of this concept [8]. Importantly, using a DNA probe containing the Sox2 binding consensus sequences, the Sox2-DNA binding was readily detectable in RR cells, but this interaction was virtually absent in RU cells. Based on these findings, we hypothesized that the key mechanistic difference between RU and RR lies with post-translational modifications of the Sox2 protein.

In this study, we tested the hypothesis that post-translational modifications of Sox2 contributes to the differential activity of Sox2 between RU and RR cells, as well as the phenotypic differences between these two cell populations. Our studies had led us to identify that Sox2 is preferentially phosphorylated in RR cells. We also identified threonine 116 as one of the Sox2 phosphorylation sites and thus, we believe that detection of phosphorylated-Sox2^T116^ holds the potential of serving as a potential marker of stem-like cells in estrogen receptor-positive BC.

## 2. Materials and Methods

### 2.1. Cell Lines and Reagents

MCF7 and ZR751 cell lines were purchased from American Type Culture Collection (ATCC, Rockville, MD, USA). Both cell lines have been authenticated using short tandem repeat DNA profiling (from TCAG Genetic Analysis Facility, Toronto, ON, Canada). These two cell lines were virally infected twice with either the mCMV or SRR2 (Sox2 regulatory region 2) reporter, and RU and RR cells were then purified as previously described [8]. The generated RU and RR cell clones were maintained in high glucose Dulbecco’s Modified Eagle Medium (DMEM) (Life Technologies, Grand Island, NY, USA) supplemented with 10% fetal bovine serum (FBS) (Life Technologies, Grand Island, NY, USA) and 10 µg/mL puromycin (Life Technologies, Grand Island, NY, USA). H_2_O_2_ (Fisher Scientific, Ottawa, ON, Canada) was freshly prepared for each experiment.

### 2.2. Primary Breast Cancer Cells, Lentiviral Transfection and Fluorescence-Activated Cell Sorting (FACS)

All primary tumors were diagnosed at the Cross Cancer Institute (Edmonton, AB, Canada). The use of these tissues has been approved by our Institutional Ethics Committee. All archival tissues were formalin-fixed and paraffin-embedded. All fresh tumor samples were processed immediately after surgery to isolate primary BC cells using a protocol described previously [9]. Briefly, tumors were cut into small pieces of 2–4 mm in the greatest dimension, which were then incubated with RPMI supplemented with Enzyme H, Enzyme R and Enzyme A (Macs Miltenyi Biotec, Auburn, CA, USA) in MACS C-tubes. Tissue shearing was performed by providing three pulses every 30 min at 37 °C. Cells were collected after centrifugation at 300× *g* for 7 min. The subsequent steps of BC isolation were based on the manufacturer’s instructions (Cancer Cell Isolation kit, Panomics, Redwood, CA, USA). After culturing for 1–2 days, cells were infected with lentivirus containing either mCMV or the SRR2 reporter. Infection was repeated twice (24 h apart) and cells were sorted into RU or RR cells approximately 48 h later, based on the green fluorescence protein (GFP) expression [9].

### 2.3. Mammosphere Formation Assay and Limiting Dilution Mammosphere Assay and Luciferase Reporter Assay

For mammosphere assay, cells were seeded and cultured as previously described [11]. Briefly, cells were trypsinized and passed through a 40 μm cell strainer (BD, Franklin Lakes, NJ, USA) and seeded into ultra-low adherent plates (Corning, NY, USA) in Mammocult media (StemCell Technologies, Vancouver, BC, Canada) as per manufacturer’s instructions. Mammosphere larger than 60 µm were counted 5–7 days after seeding. Limiting dilution assay has been used as a gold standard for the assessment of CSCs [12,13]. In brief, cells were seeded in 96-well low-adherent plate (Corning, NY, USA) at 10 limiting dilutions ranging from 1 to 400 cells. Each dilution had 6 replicates, and each well was scored for presence or absence of mammosphere after 5–7 days. Data were analyzed using the Extreme Limiting Dilution Analysis (ELDA) software for three independent experiments [14]. Luciferase reporter assay was performed using luciferase assay system kit (#E4530, Promega, Corporation, Madison, WI, USA) according to the manufacturer’s protocol, plated on Costar white polystyrene opaque 96-well plates (#3912, Corning, NY, USA) and analyzed on the FLUOstar Omega multi-mode microplate reader (BMG Labtech, Ortenburg, Germany).

### 2.4. Mass-Spectrometry Analysis and Database Search

RR and RU cells derived from MCF7 were transfected with a flag-tagged-*SOX2* vector. Sox2 binding proteins were captured using anti-flag M2 affinity beads according to the manufacturer’s suggestion (Sigma, Oakville, Ontario, Canada). Briefly, cell lysates derived from MCF7 cells transfected flag-tagged-*SOX2* were incubated with anti-flag M2 affinity beads (Sigma) at 4 °C overnight. The beads were washed by Tris-buffered saline (TBS) (Sigma) three times. Sox2 proteins were eluted using 0.1 M glycine HCl, pH 3.5 (Sigma) and then subjected to tryptic digestion [15]. The tryptic peptide mixtures were analyzed by mass spectrometric analysis using a Q-TOF Premier mass spectrometer (Waters, Milford, MA, USA) equipped with a nanoACQUITY Ultra Performance LC system (Waters) as previously described [16]. Protein identification was performed using the Mascot 2.2 search engine (Matrix Science, Boston, MA. USA) for searching the Swiss-Prot database (version 57.4, 410, 518 sequences). Searching was restricted to *Homo sapiens* and performed using the following parameters: fixed modification, carbamidomethyl (cys); variable modifications, oxidation (Met) and phosphorylation on serine, threonine, or tyrosine; missed cleavages: 1; peptide tolerance: 30 ppm; MS/MS tolerance: 0.2 Da; Peptide charge: 1+, 2+ and 3+. All the identified peptides were above the Mascot threshold score for identity with a confidence level of >95%. Each experiment consists of a negative control sample (cells without transfection) and an experimental sample. For each sample, the peptide mixture was analyzed with five consecutive runs, with each run carried out using an optimal and maximized sample loading; peptide precursor ion exclusion strategy was applied to exclude relatively high abundance peptides identified from the previous runs, thus allowing the identification of relatively lower abundance peptides [17,18].

### 2.5. Antibody Production and Purification

The mouse monoclonal antibody (mAB) production was performed by Genescript USA, Inc. (Piscataway, NJ, USA). In brief, phosphorylated peptide (CKYRPRRK (PTHR) KTLMKK) was conjugated with keyhole limpet hemocyanin (KLH). 10 BALB/c (Bagg albino) mice were immunized with conjugated peptide. Mice which showed satisfactory immune response were selected for further hybridoma production step. mAB reactivity and antibody titer was determined by Genscript USA, Inc. by performing enzyme-linked immunosorbent assay (ELISA) with Sox2 and pSox2 peptide used as coating antigens. Once reactivity was confirmed, 5 mL of each hybridoma cell culture supernatant (with 0.02% sodium azide preservative) was lyophilized. The lyophilized powder was stored at −20 °C and reconstituted in distilled deionized water prior to use. After screening by immunoprecipitation and reverse immunoprecipitation, 4C7G2 was selected as the best of 6 clones received from Geneescript. Large-scale mAB production was then performed by inducing ascite formation in BALB/c mice followed by affinity purification (Genscript). Final confirmation of the immune-reactivity of 4C7G2 was performed using indirect ELISA, with Sox2 and pSox2^T116^ peptide used as coating antigens, and a goat anti-mouse IgG peroxidase conjugated secondary antibody.

### 2.6. Immunoprecipitation, Peptide Dot Blotting and Western Blotting

All immunoprecipitation (IP) experiments were carried out using all received 6 clones: 4C7G2, 4C7G6, 4C7H7, 4C7A4, 4C7A5 and 4C7D8 (2 µg) with 500 µg cell lysate collected in ice-cold RIPA buffer (Cell Signaling Technologies, Danvers, MA, USA) supplemented with protease and phosphatase inhibitors (EMD Millipore, Etobicoke, ON, Canada) as described previously [19]. Peptide dot blotting was carried out using phospho-Sox2^T116^ specific immunizing peptide and the unphopshporylated Sox2 peptide as follows 0.05, 0.1, 0.2, 0.5, 1 and 2 µg peptide diluted in PBS was directly pipetted onto nitrocellulose membrane and allowed to dry. The membrane was then blocked in 5% BSA TBS 0.05% Tween 20 for 1 h, and incubated overnight with mAB 4C7G2. Protein was detected using Pierce ECL Western blotting substrate.

### 2.7. Immunohistochemistry

Immunohistochemistry was performed using 4C7G2 (pSox2^T116^) in 35 archival BC tumor samples. In brief, to detect pSox2^T116^, antigen retrieval (15 min) was done using pre-heated Citrate buffer (pH = 6.0). The subsequent immunohistochemistry was performed using standard techniques and the pSox2^T116^ antibody (1:500) [20]. Two pathologists scored the proportion of positively stained tumor cells and the staining intensity independently, and a consensus score was given for each case. pSox2^T116^ staining was detected mainly in the nucleus. One of the characteristics of pSox2^T116^ staining is that strongly positive tumors cells typically formed small clusters of 10–20 cells, and these clusters scattered throughout the tissue sections. Thus, for the evaluation of pSox2^T116^ staining, the entire whole tumor sections were initially screened for these foci at 100× magnification. For each positive focus, the proportions (0–100%) of negative cells, weakly positive cells and strongly positive cells were estimated at 200× magnification. Up to five positive foci were assessed. In the event that positive foci were less than 5 in the entire tissue sections, areas containing high proportions of weakly positive cells were preferentially chosen for scoring. For the calculation of the final scoring, the proportion of strongly positive cells carried a weight of 2 whereas that of weakly positive cells carried a weight of 1. Tumors with an average score of ≥20 in the 5 fields evaluated were considered positive whereas those with an average score of <20 in the 5 fields evaluated were considered negative.

### 2.8. Statistical Analysis

All of the analysis was performed by using SPSS (11.5) software. The association between the pSox2^T116^ expression and clinicopathological parameters was performed by χ^2^ or Fisher’s exact test. A *p* value < 0.05 was considered significant.

## 3. Results

### 3.1. RR Cells Are More Stem-Like than RU Cells

**Figure 1:**
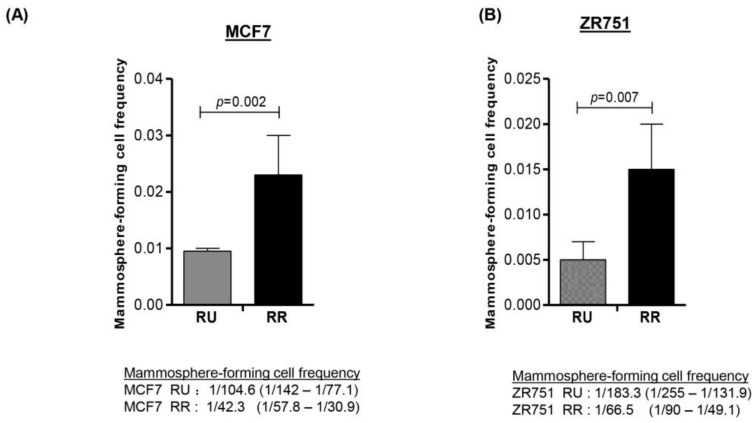
Reporter responsive (RR) cells were significantly more capable in forming mammospheres in the setting of limiting dilutions. Reporter unresponsive (RU) and RR cells derived from MCF7 and ZR751 cells were serially diluted and seeded into low attachment 96-well plates. After 7 days, the spheres were counted and data was analyzed using ELDA software. (**A**) RR cell subset purified from MCF7 cells had a median frequency of 1/42.3 mammosphere forming cells, as compared to 1/104.6 in the RU subset (*p* = 0.002); (**B**) RR cell subset purified from ZR751 cells had a median frequency of 1/66.5 mammosphere forming cells, as compared to 1/183.3 mammosphere forming cells in the RU subset (*p* = 0.007).

We have previously demonstrated that RR cells derived from MCF7 and ZR751 cells are more tumorigenic and stem-like than RU cells [8]. Furthermore, the phenotypic differences between these two cell subsets is dependent on Sox2, since siRNA knockdown of Sox2 in RR cells effectively abrogated the RR-associated phenotype [8]. In this study, we further substantiated the link between the RR phenotype and cancer stemness by performing limiting dilution-mammosphere assay. As shown in Figure 1A, we found that RR cells derived from MCF7 contained a significantly higher frequency of mammosphere-forming cells than RU cells (1/42.3 versus 1/104.6, *p* = 0.002). Similar results were obtained with ZR751 cells (1/66.5 versus 1/183.3, *p* = 0.007) (Figure 1B). These findings support the concept that RR cells are more stem-like than RU cells.

### 3.2. Phosphorylation of Sox2 Is Substantially Higher in RR Cells

**Figure 2:**
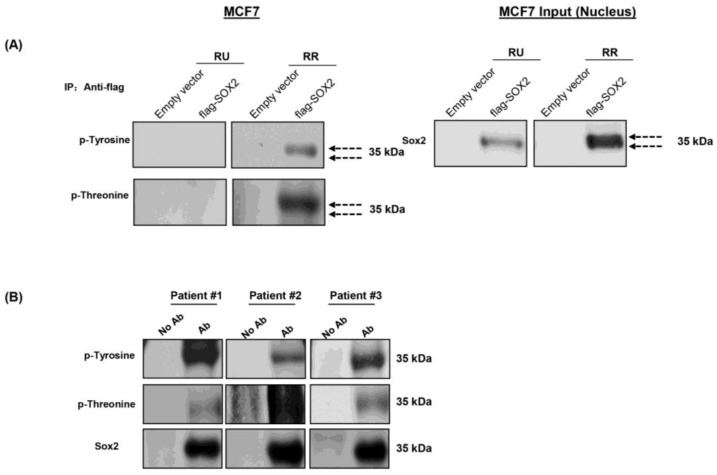
RR cells had higher phosphorylation of Sox2. Immunoprecipitation experiments were carried out using RU and RR cells derived from MCF7 cells transfected with flag-*SOX2*. Precipitation was carried out using the anti-flag M2 affinity gel and Western blot was carried out using Sox2, p-Tyrosine and p-Threonine antibodies. (**A**) Right panel represents immunoprecipitation of flag-Sox2 pulled down in both RU and RR cells derived from MCF7 cells. Of note, two bands of Sox2 were identified in RR cells whereas only one band was identified in RU cells. Left panel represents Western blots results probed with anti-phospho-tyrosine (p-tyrosine) and anti-phospho-threonine (p-threonine) revealed evidence of Sox2 phosphorylation in RR but not RU cells; (**B**) Western blot of Sox2 was performed using 3 patient tumor samples. Immuno-reactivity with anti-*p*-tyrosine and anti-*p*-threonine was detected in all 3 samples.

Since the expression level of Sox2 was found to be similar between RU and RR cells [9], we hypothesized that post-translational modifications of Sox2 may be an important factor contributing to the RU/RR dichotomy. To search for evidence of post-translational modifications of Sox2, we performed immunoprecipitation experiments using purified RU and RR cells derived from MCF7. To increase the robustness of our assay, we transfected a flag-tagged-*SOX2* vector into these cells, and the flag-Sox2 protein was pulled down using the anti-flag M2 affinity gel. As shown in Figure 2A, appreciable amounts of Sox2 were pulled down in both RU and RR cells, although there were two distinct Sox2 bands in RR cells as compared to only one band in RU cells. Correlating with this finding, probing with antibodies reactive with anti-phospho-tyrosine and anti-phospho-threonine antibodies highlighted the top Sox2 band in RR cells; no detectable signal was found in RU cells. To ensure that phosphorylation of Sox2 is not a cell-line specific phenomenon, we examined Sox2 phosphorylation in fresh primary BC tumor samples. As shown in Figure 2B, immunoblots prepared from 3 of 3 estrogen receptor-positive BC samples were reactive with anti-phospho-tyrosine and anti-phospho-threonine. Due to the relatively small number of tumor cells extractable from these patient samples, we were unable to purify sufficient RU and RR cells for immunoprecipitation experiments. Taken together, we found that Sox2 is preferentially phosphorylated in RR but not RU cells, and this finding led us to speculate that the lack of Sox2 phosphorylation in RU cells may explain why Sox2 fails to bind to and activate the SRR2 reporter in this cell subset.

### 3.3. Sox2 Is Phosphorylated at T116 in RR But Not RU Cells

**Figure 3:**
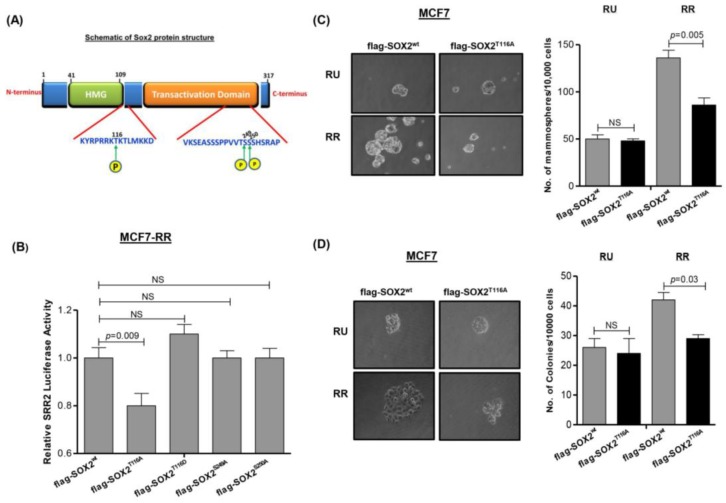
Phosphorylation of Sox2 at T116 contributes to RR phenotype. (**A**) Schematic of Sox2 protein structure and potential phosphorylation sites; HMG=high mobility group domain (**B**) Transfection of the flag-*SOX2*^T116A^ mutant into RR cells derived from MCF7 cells significantly decreased the SRR2 luciferase reporter activity as compared to the cells transfected with the flag-*SOX2^wt^* vector. In comparison, transfection of three other Sox2 mutants (flag-*SOX2*^S249A^, flag-*SOX2*^S250A^ and flag-*SOX2*^T116D^) did not result significant changes in the SRR2 luciferase reporter activity; (**C**) Transfection of flag-*SOX2*^T116A^ mutant into RR cells derived from MCF7 cells resulted in a significantly lower mammosphere formation capability compared to cells transfected with flag-*SOX2^wt^* vector. In RU cells, the mammosphere formation capability was relatively low, and transfection of flag-*SOX2*^T116A^ mutant did not result in appreciable changes; (**D**) Transfection of the flag-*SOX2*^T116A^ mutant into RR cells derived from MCF7 cells resulted in a significantly lower methylcellulose colony formation, compared to cells transfected with flag-*SOX2^wt^* vector.

We next sought to identify specific phosphorylation site(s) of Sox2 in RR cells. Using various computer software programs including Phospho-Motif finder, Net-phos 2.0 and ELM, we determined that 8 of 41 serine residues at TAD (transactivation domain) and 2 of 14 threonine residues at HMG (high mobility group domain) are potential Sox2 phosphorylation sites, with a confidence level of 95% (illustrated in Figure 3A). We then employed liquid chromatography-mass spectrometry analysis (LC-MS) to analyze flag-Sox2 purified from RR cells. As shown in Supplementary Figure S1, our analysis revealed evidence that threonine 116 (T116) of Sox2 is a phosphorylation site, and this signal was detectable in RR but not RU cells.

### 3.4. Phosphorylation of Sox2^T116^ Is Important in Conferring SRR2 Reporter Activity and Stem-Like Features

To evaluate the functional significance of Sox2 phosphorylation at T116, we employed site-directed mutagenesis to generate a mutant with alanine replacing threonine at T116, namely Sox2^T116A^. As shown in Figure 3B, transfection of *SOX2^T116A^* into RR cells derived from MCF7 resulted in a significant decrease in the SRR2 reporter luciferase activity, one of the read-outs for the SRR2 reporter (*p* = 0.003). In comparison, transfection with *SOX2^T116D^* (threonine→aspartic acid at residue 116, designed to mimic T116 phosphorylation), *SOX2^S249A^* (serine→alanine at residue 249) or *SOX2^S250A^* (serine→alanine at residue 250) did not result in any significant change in the SRR2 reporter luciferase activity. As expected, no significant change in the luciferase activity was seen when the same experiment was repeated using RU cells (not shown).

Next, we performed methylcellulose colony formation and mammosphere formation assays to examine the functional importance of Sox2^T116^ phosphorylation. RR cells derived from MCF7 transfected with flag-*SOX2* were compared to those transfected with flag-*SOX2^T116A^* mutant. As shown in Figure 3C,D, compared to cells transfected with flag-*SOX2*, cells transfected with flag-*SOX2^T116A^* showed a significant decrease in methylcellulose colony formation and mammosphere formation assay (*p* = 0.005 and *p* = 0.03, respectively).

### 3.5. Generation and Characterization of Anti-pSox2^T116^ Antibody

**Figure 4:**
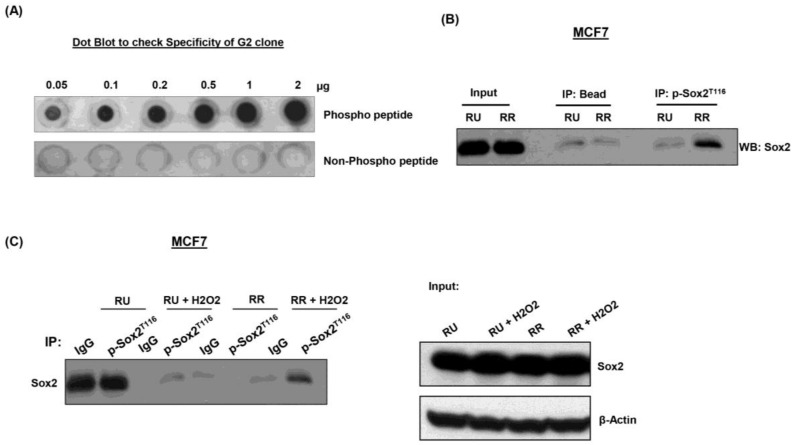
Acquisition of Sox2T116 phosphorylation occurs upon oxidative stress. (**A**) Dot blot experiment was performed and results showed that G2 antibody was reactive with pSox2^T116^ in a concentration-dependent manner. Furthermore, G2 had no immunoreactivity toward the unphosphorylated Sox2 peptide; (**B**) Immunoprecipitation experiments were performed and G2 clone pulled down Sox2 protein in RR but not RU cells derived from MCF7 cells. Input shows an equal amount of Sox2 protein was expressed in RU and RR cells; (**C**) Immunoprecipitation experiment was performed using RU and RR cells derived from MCF7 cells with or without exposure to oxidative stress (H_2_O_2_). RU cells ‘acquired’ Sox2 phosphorylation at T116 after oxidative stress.

To further characterize the significance of Sox2 phosphorylation at T116, we generated monoclonal antibodies against this epitope. Using ELISA, we found that antibodies from 6 clones showed relatively high immunoreactivity for the phospho-Sox2^T116^ peptide, relative to the corresponding unphosphorylated Sox2 peptide (data not shown). Antibodies generated from these 6 hybridomas were then used to probe Western blots prepared from RU and RR cells derived from MCF7 cells. As illustrated in Supplementary Figure S2, clone G2 showed the most definitive distinction between RR and RU cells, with RR but not RU cells showing immunoreactivity towards these two antibodies. To further demonstrate that the G2 clone is specific for phospho-Sox2^T116^, dot blot experiments were performed. As shown in Figure 4A, clone G2 showed immunoreactivity to the phospho-Sox2^T116^ peptide in a concentration-dependent manner; in contrast, no signal was observed with the unphosphorylated Sox2 peptide. We also performed immunoprecipitation experiment to validate the G2 clone, with the immunoprecipitation step carried out using the G2 clone and Western blotting carried out using a commercially available anti-Sox2 antibody. As shown in Figure 4B, we found that the expression of phospho-Sox2^T116^ was largely restricted to RR cells.

### 3.6. pSox2^T116^ Is Acquired during the RU/RR Conversion Upon Oxidative Stress

We have recently found that RU cells derived from MCF7 and ZR751 can be converted to RR cells under oxidative stress generated by H_2_O_2_ [21]. Importantly, we found that converted RR cells are more stem-like and tumorigenic compared to native RU cells [21]. Thus, we asked if converted RR cells have phosphorylated Sox2 at T116 that is coupled with its binding to SRR2. As shown in Figure 4C, we found that converted RR cells “acquired” phospho-Sox2^T116^ expression. In our previous publication [11], we found that Sox2 binds to the SRR2 consensus sequences substantially stronger after oxidative stress, and this finding correlated with the acquisition of phospho-Sox2^T116^.

Furthermore, we also have shown that converted RR cells derived had significantly higher expressions of cancer stemness-related genes such as PROM1, GPR49 and MUC15 as compared to the native RU cells [21], which again validate our hypothesis that Sox2 phosphorylation at T116 regulates cancer stem-like features.

### 3.7. Immunohistochemical Studies of pSox2^T116^ in Primary BC Tumors

**Figure 5:**
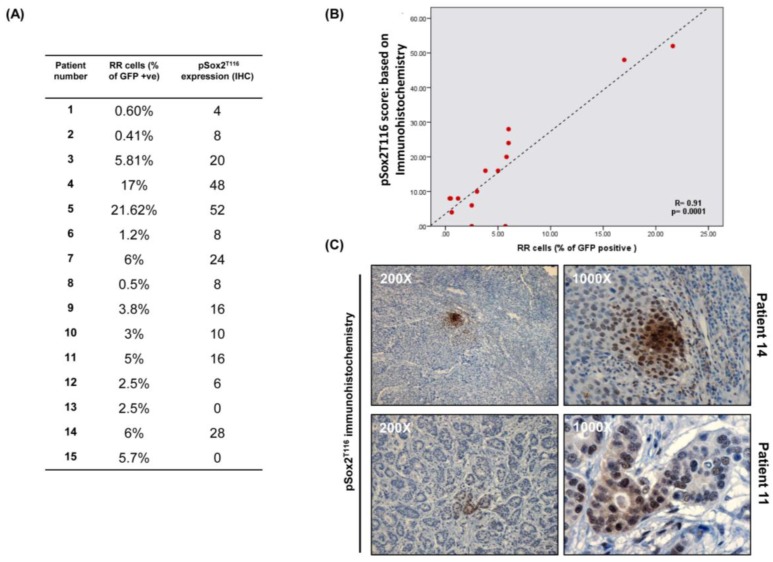
Immunohistochemical studies of pSox2^T116^ in primary BC tumors. (**A**) 15 primary BC tumors showing % of RR cells detected by flow analysis and pSox2^T116^ expression detected by immunohistochemistry; (**B**) Spearman’s rank correlation was carried out between % of RR cells detected by flow analysis and pSox2^T116^ expression detected by immunohistochemistry. Positive correlation was observed between % of RR cells and pSox2^T116^ expression (R = 0.91; *p* = 0.0001); (**C**) Two representative images have been shown here for pSox2^T116^ immunohistochemistry.

We then tested if our generated antibody reactive with phospho-Sox2^T116^ can be used as a surrogate marker to identify RR cells in primary BC tumors. To this end, we performed immunohistochemistry in 15 randomly chosen, primary BC tumors. The percentage (%) of tumor cells expressing phospho-Sox2^T116^ was correlated with that of RR cells detectable by flow cytometry. As shown in Figure 5A,B, we found a significant positive correlation between % of cells showing immunohistochemical reactivity with anti-phospho-Sox2^T116^ and % of RR cells detectable by flow cytometry (*p* = 0.0001). As illustrated in Figure 5C, phospho-Sox2^T116^ immunostaining was largely confined to small subsets of cancer cells; the majority of the tumor cells were negative for this marker. Interestingly, phospho-Sox2^T116^-positive cells often clustered in small foci, with the staining intensity gradually decreased with increasing distance from the center of these foci.

We then evaluated the clinical significance of phospho-Sox2^T116^ expression in BC by expanding our cohort to 35 cases. As summarized in Table 1, tumors with relatively high % of phospho-Sox2^T116^-positive tumor cells were significantly more likely to have high histological grade (*p* = 0.006) and intra-tumoral vascular/lymphatic involvement (*p* = 0.001). Moreover, tumors with a high % of phospho-Sox2^T116^-positive cells were also more likely to express the estrogen receptor (*p* = 0.032). However, we did not identify significant correlation between tumors with a high % of phospho-Sox2^T116^-positive cells with patient age, mitotic rate, lymph node metastasis, architecture and the progesterone receptor status.

## 4. Discussion

We have previously revealed a novel phenotypic dichotomy in estrogen receptor-positive BC cell lines and patient samples, with RR cells being significantly more tumorigenic and stem-like than RU cells [8,9]. Similar findings were subsequently described and published by other groups [6,7]. Since Sox2 is essential to this phenotypic dichotomy, and the fact that RU and RR cells do not differ substantially in the Sox2 protein expression and subcellular distribution, we hypothesized that post-translational modification of the Sox2 protein is a key contributory factor to the RU/R dichotomy. Our experimental data strongly supports this concept based on the following lines of evidence. First, Sox2 was found to be phosphorylated in RR but not RU cells derived from cell lines and primary patient samples. With the finding that Sox2^T116^ is one of the phosphorylation sites, we have presented evidence that phospho-Sox2^T116^ exists in RR but not RU cells. Second, we found that Sox2^T116A^ mutant carry dominant negative effects, exerting inhibition to the tumorigenic/stem-like phenotype of RR cells. Third, the expression of phospho-Sox2^T116^ was appreciably increased in RU cells exposed to oxidative stress, a stimulation that has been shown to raise the level of the RR phenotype in RU cells [21]. In keeping with our concept that phospho-Sox2^T116^ is a marker of RR cells, the small subset of cells carrying stem-like features, tumor cells carrying this epitope were found to constitute a small percentage of tumor cells in BC.

Sox2 is an important embryonic stem cell marker, and it has been shown to promote cancer stemness in a large number of human cancer models, including breast [22], gastric [23], ovarian [24], prostate [25] and lung [26]. The direct role of Sox2 in conferring cancer stemness has been nicely illustrated by many studies. For instance, siRNA knockdown of Sox2 in the CSCs population in lung cancer was found to significantly decrease tumor growth and metastases in in vivo xenograft model [27]. Similarly, knockdown of Sox2 was found to efficiently inhibit in vitro sphere formation and reduced tumor formation in glioblastoma and gastric cancer, respectively [23,28]. Furthermore, the EGFR-induced enhancement of the self-renewal capacity in prostate cancer cells correlates with an upregulation of Sox2 [25]. Lastly, knockdown of Sox2 using siRNA in head and neck cancer was found to result in a down-regulation of ABCG2, a CSCs marker known to be associated with chemo-resistance [29]. In BC research, it was found that BC cells harvested from mammospheres formed by primary tumor cells or MCF7 have a higher level of Sox2 expression, and siRNA knockdown of Sox2 inhibited mammosphere formation [22]. Correlating with the biological importance of Sox2 in conferring cancer stemness and tumorigenecity in BC, multiple studies have shown that the protein expression of Sox2 detectable by immunohistochemistry significantly correlates with various clinicopathologic parameters. For instance, Sox2 expression was found to significantly correlate with a high frequency of disease recurrence and short disease-free survival [30]. In another study, it was found that Sox2 expression correlates with high histologic grade, large tumor size and a high proliferation index [31]. Furthermore, Sox2 expression was detected in 28% of invasive breast carcinoma and 44% in ductal carcinoma [32]. Similarly, Leis et al. [22] observed Sox2 expression in 158 BC patient samples and demonstrate that Sox2 expression was significantly higher at early stage of the disease indicating Sox2 expressed in the initial stage of tumor progression.

There is evidence that the biological function of Sox2 is regulated by its post-translational modifications. Highly relevant to this current study, it has been shown that Sox2 phosphorylation plays a pivotal role in regulating its biological functions in mouse embryonic stem cells. Specifically, it has been observed that AKT directly interacts with Sox2, promotes its stabilization by promoting its phosphorylation at T118 (which is equivalent to T116 in humans) and enhances its transcriptional activity in this cell type [33]. Recently Schaefer et al. [34] also reported that the AKT/Sox2 molecular axis regulates clonogenicity in BC. Furthermore, phosphoproteome analysis has identified serine 249, serine 250 as potential Sox2 phosphorylation sites [35]. In a recent study, it was found that PKCI phosphorylates Sox2 at T118 site and drives tumorigenecity and cancer stem-like features in human lung squamous cell carcinoma cells [36]. These findings are in keeping with our model that Sox2 phosphorylation is an important mechanism in regulating the observed intra-tumoral heterogeneity (i.e., RU/RR dichotomy), and by extension, the level of cancer stemness and tumorigenecity of individual cells in cell lines and tumors. While it is evident that Sox2 phosphorylation is important in regulating the transcriptional activity of Sox2 and its ability to confer cancer stemness, it remains to be determined which Sox2 residue(s) are the critical sites. In one of our studies using various Sox2 mutants with mutations involving T116 and/or T118, we found that both sites are functionally important in ALK-positive anaplastic large-cell lymphoma (ALK + ALCL) cells (manuscript in preparation). Recently, Fang *et al*. also reported that Sox2 phosphorylation by AKT1 regulates Sox2 stability in embryonic stem cells and plays an important role during differentiation [37]. We have recently performed siRNA screen and we have identified a number of potential kinases that might be responsible for the phosphorylation of Sox2; these results are currently being validated.

One of the important aspects of this current study is highlighted by the observation that Sox2 phosphorylation can be modulated in response to oxidative stress, a stimulus previously shown to increase cancer stemness [38]. In this regard, we recently published that RU cells derived from MCF7 and ZR751 can be induced to convert into RR cells under oxidative stress, and converted RR cells were confirmed to demonstrate significant increases in cancer stem-like features and tumorigenecity *in vitro* and *in vivo* [21]. The observation that the level of phospho-Sox2^T116^ in RU cells stimulated with oxidative stress further suggests that this post-translational change of Sox2 is an integral part in shaping the RR phenotype, and not merely an association. Interestingly, the level of phospho-Sox2^T116^ in RR cells also increased upon oxidative stress, although the increment was not as dramatic as that of RU cells. This finding supports the concept that the level of cancer stemness can be ‘tuned’ to the environment at the cellular level, and cancer stemness is not an all-or-none phenomenon.

The generation and availability of a mAB reactive with anti-phospho-Sox2^T116^ has greatly advanced our understanding of the role of Sox2 in BC and the clinical significance of the RU/RR dichotomy. Our pSox2^T116^ immunohistochemistry result brings forth the novel idea to identify the cancer stem-like cells in BC. Interestingly, a significant and positive correlation between RR% by flow analysis and pSox2^T116^ expression has validated our findings. As we have shown that phosphorylation of Sox2 at threonine 116 is directly linked to Sox2 function, screening of BC for pSox2^T116^ staining may have significant implications for cancer treatment. Our collected data have broad implications for the development of new therapeutic strategies to target cancer stem-like cells in BC.

## 5. Conclusions

All together, these results indicate that either cancer stem-like cells or converted cancer stem-like cells have higher pSox2^T116^ expression and identification of these cells by immunohistochemistry can be used as a new potential marker to identify the cancer-initiating population in BC patients. Furthermore, for the first time, we have developed and characterized a novel monoclonal antibody against Sox2 phosphorylation at T116 site, which can be used as a surrogate potential marker to identify more tumorigenic and cancer stem-like subpopulation in BC. The novelty of employing pSox2^T116^ as a potential marker in BC will allow us to functionally identify very small cell subsets, responsible for relapse in future, rather than to rely on the endpoint expression of other proteins.

## Figures and Tables

**Table 1:** Correlation between pSox2T116 expression and clinicopathological features in breast cancer.

Characteristics	pSox2^T116^ Expression			
	Low Expression n (%)	High Expression n (%)	Total n	*p* Value
Age				0.691
<50	5 (55.6%)	4 (44.4%)	9	
≥50	17 (65.4%)	9 (34.6%)	26	
Mitotic				
1	12 (75%)	4 (25%)	16	0.293
2	1 (33.3%)	2 (66.7%)	3	
3	9 (56.2%)	7 (43.8%)	16	
Histological grade				
Grade 1	8 (72.7%)	3 (27.3%)	11	0.006
Grade 2	7 (100%)	0	7	
Grade 3	7 (41.2%)	10 (58.8%)	17	
Blood/Invasion				
No	19 (86.4%)	3 (13.6%)	22	0.001
Yes	3 (23%)	10 (76.9%)	13	
pN				
1	2 (66.7%)	1 (33.3%)	3	0.332
2	12 (75%)	4 (25%)	16	
3	8 (50%)	8 (50%)	16	
Architecture				
1	2 (100%)	0	2	0.371
2	5 (62.5%)	3 (37.5%)	8	
3	15 (60%)	10 (40%)	25	
ER status				
Negative	14 (82.4%)	3 (17.6%)	17	0.032
Positive	8 (44.4%)	10 (55.6%)	18	
PR status				
Negative	8 (61.5%)	5 (38.5%)	13	0.591
Positive	14 (63.6%)	8 (36.4%)	22	

## Supplementary Materials

The following are available online at http://www.mdpi.com/2072-6694/10/2/41/s1, Figure S1: Mass spectrometric analysis of Sox2 proteins. By LC-MS, phosphorylation of Sox2 at T116 was found only in MCF7RR cells. A representative spectrum of phosphopeptide (peptide sequence: Kp(T)KTLMK from Sox2 has been shown here. Figure S2: Identification of the monoclonal antibodies produced in the hybridoma supernatant of different cell clones. Western blot was performed in RU and RR cells derived from MCF7 cells to identify the clone showing the definitive distinction between RR and RU cells and, as indicated, G2 clone showed the most definitive distinction with RR but not RU cells.
