# Supplementary material for: Phosphorylation of Sox2 at Threonine 116 is a Potential Marker to Identify a Subset of Breast Cancer Cells with High Tumorigenecity and Stem-Like Features
Source: Cancers (Basel). 2018 Feb 3;10(2):41. doi: 10.3390/cancers10020041 (PMC5836073; doi:10.3390/cancers10020041)
Supplement: Supplementary file 1 [file cancers-10-00041-s001.pdf]

Supplementary Materials: Phosphorylation of Sox2 at Threonine 116 is a Potential Marker to Identify a Subset of Breast Cancer Cells with High Tumorigenecity and Stem-Like Features

Nidhi Gupta, Keshav Gopal, Chengsheng Wu, Abdulraheem Alshareef, Alexandra Chow, Fang Wu, Peng Wang, Xiaoxia Ye, Gilbert Bigras and Raymond Lai

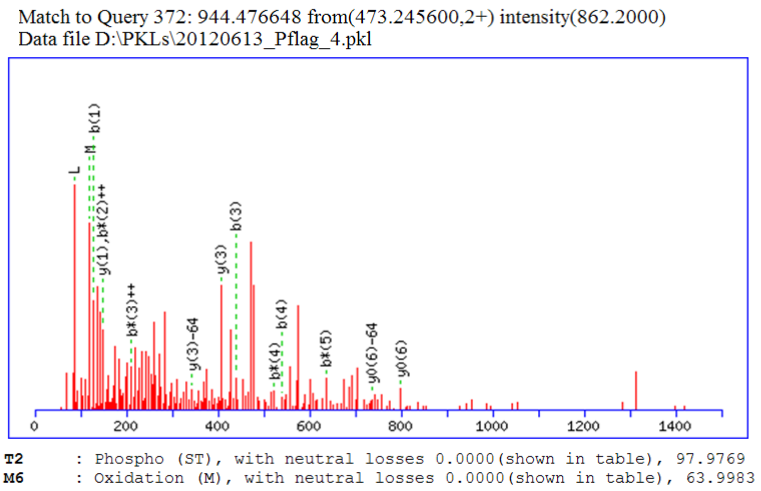

Figure S1. Mass spectrometric analysis of Sox2 proteins. By LC-MS, phosphorylation of Sox2 at T116 was found only in MCF7RR cells. A representative spectrum of phosphopeptide (peptide sequence: Kp(T)KTLMK from Sox2 has been shown here;

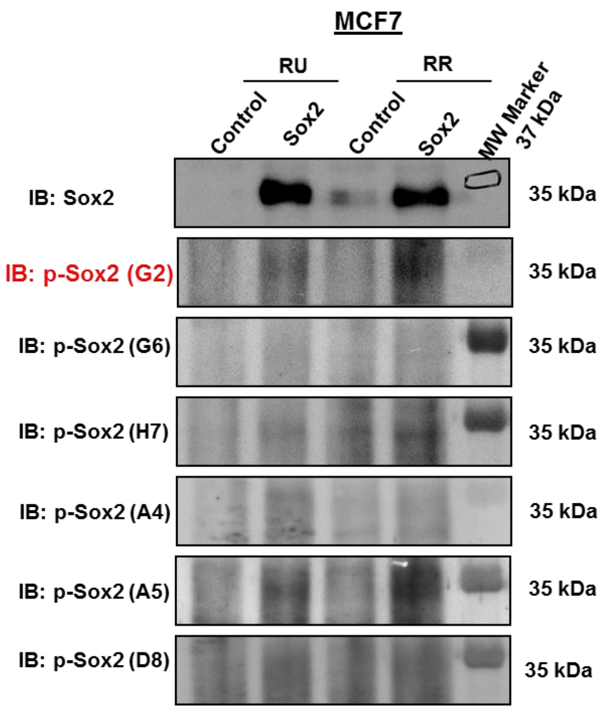

Figure S2. Identification of the monoclonal antibodies produced in the hybridoma supernatant of different cell clones. Western blot was performed in RU and RR cells derived from MCF7 cells to identify the clone show the definitive distinction between RR and RU cells and as indicated G2 clone showed the most definitive distinction with RR but not RU cells.

© 2018 by the authors; licensee MDPI, Basel, Switzerland. This article is an open access article distributed under the terms and conditions of the Creative Commons by Attribution (CC-BY) license (http://creativecommons.org/licenses/by/4.0/)
